# Assembly of the peripheral stalk of ATP synthase in human mitochondria

**DOI:** 10.1073/pnas.2017987117

**Published:** 2020-11-09

**Authors:** Jiuya He, Joe Carroll, Shujing Ding, Ian M. Fearnley, Martin G. Montgomery, John E. Walker

**Affiliations:** ^a^Medical Research Council Mitochondrial Biology Unit, University of Cambridge, Cambridge Biomedical Campus, CB2 0XY Cambridge, United Kingdom

**Keywords:** human mitochondria, ATP synthase, assembly, peripheral stalk

## Abstract

The adenosine triphosphate (ATP) synthase in human mitochondria is a membrane bound assembly of 29 proteins of 18 kinds organized into F_1_-catalytic, peripheral stalk (PS), and c_8_-rotor ring modules. All but two membrane components are encoded in nuclear genes, synthesized on cytoplasmic ribosomes, imported into the mitochondrial matrix, and assembled into the complex with the mitochondrial gene products ATP6 and ATP8. Intermediate vestigial ATPase complexes formed by disruption of nuclear genes for individual subunits provide a description of how the various domains are introduced into the enzyme. From this approach, it is evident that three alternative pathways operate to introduce the PS module (including associated membrane subunits e, f, and g). In one pathway, the PS is built up by addition to the core subunit b of membrane subunits e and g together, followed by membrane subunit f. Then this b-e-g-f complex is bound to the preformed F_1_-c_8_ module by subunits OSCP and F_6_. The final component of the PS, subunit d, is added subsequently to form a key intermediate that accepts the two mitochondrially encoded subunits. In another route to this key intermediate, first e and g together and then f are added to a preformed F_1_-c_8_-OSCP-F_6_-b-d complex. A third route involves the addition of the c_8_-ring module to the complete F_1_-PS complex. The key intermediate then accepts the two mitochondrially encoded subunits, stabilized by the addition of subunit j, leading to an ATP synthase complex that is coupled to the proton motive force and capable of making ATP.

## Significance

The production of ATP in mitochondria requires the oxidation of energy rich compounds to generate a proton motive force (pmf), a chemical potential difference for protons across the inner membrane. This pmf powers the ATP synthase, a molecular machine with a rotary action, to synthesize ATP. The assembly of human ATP synthase from 27 nuclear encoded proteins and two mitochondrially encoded subunits in the inner organellar membrane involves the formation of intermediate modules representing the F_1_-catalytic domain, the peripheral stalk, associated membrane subunits, and the c_8_ ring in the membrane part of the rotor. Here, we describe how components of the peripheral stalk and three associated membrane subunits are assembled and introduced into the enzyme complex.

**Fig. 1.**
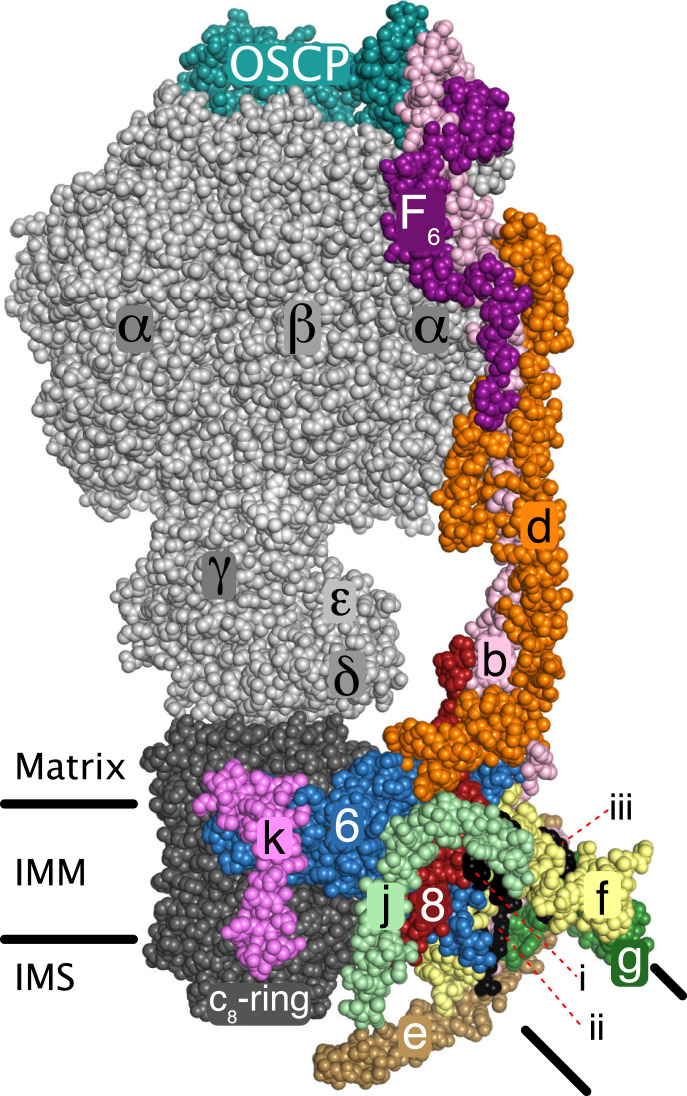
Organization of peripheral stalk and associated subunits in a monomer in dimeric ATP synthase in bovine mitochondria. The enzyme is inhibited by residues 1–60 of the inhibitor protein IF_1_, which is bound to the catalytic domain and not defined in the figure (4). Black horizontal lines represent the inner mitochondrial membrane (IMM) separating the matrix from the intermembrane space (IMS). The PS is formed from subunits OSCP, F_6_, d, ATP8 (designated “8”), and b. Subunit b has two transmembrane α-helices, residues 33–47 of bH2 and residues 55–73 of bH3 with an angle of 45° between them. The N-terminal amphipathic α-helix bH1 (residues 19–29) lies in the lipid head-group region on the matrix side of the membrane. These three α-helices form the scaffold for a wedge structure in the membrane domain to which subunits e, f, g, j, and ATP8, all with single transmembrane α-helices, are associated. Voids between proteins in the wedge are filled with five specifically bound lipids, three cardiolipins, and two other lipids, tentatively modeled as phosphatidylglycerols (4). The positions of one cardiolipin (defined as CDL1) and the two phosphatidyl glycerols (defined as LHG4 and LHG5) are indicated by *i*–*iii*, respectively. The wedge braces ATP6 against the c_8_ ring and in the dimer the two interacting wedges hold the rotatory axes of the monomeric enzymes at a range of acute angles via pivoting about the j subunits in the surfaces of the two wedges (4). Subunit j is involved in the monomer–monomer interface in yeast mitochondria (13), but the interactions are different from in the bovine structure (4). Subunits e and g make a separate domain not in contact with ATP6 (designated “6”) and subunit j, that may promote inner membrane curvature (33–35). Subunit k may be involved in tethering dimers together (5). The mitochondrial pmf drives the rotation of the c_8_ ring and the attached central stalk (subunits γ, δ, and ε) by the translocation of protons through the interface between the c_8_ ring and ATP6. The rotation of the central stalk carries energy into the catalytic sites of the three β-subunits in the F_1_ domain (subunit composition α_3_β_3_γδε).

Energy derived from oxidative metabolism generates a proton motive force (pmf) across the inner membranes of mitochondria, which the adenosine triphosphate (ATP) synthase harnesses to provide most cellular ATP from adenosine diphosphate and phosphate by a rotary catalytic mechanism (1–3). Human ATP synthase is closely related in subunit composition and sequences to the bovine enzyme (3). Bovine ATP synthase is a protein-lipid complex made from 29 protein subunits of 18 types, including a regulatory protein IF_1_, contributing a mass of 591 kDa, and five specifically bound phospholipids (Fig. 1 and *SI Appendix*, Fig. S1) (3, 4). The protein subunits of the human enzyme are organized by an assembly process into membrane extrinsic and membrane intrinsic domains, linked by a central stalk and a peripheral stalk (PS) (5, 6). The PS is often taken to denote the membrane extrinsic region of the stator. Here, because of the way this region of the enzyme is assembled, the term PS signifies the membrane extrinsic region of the peripheral stalk plus the membrane intrinsic region of subunit b and the associated supernumerary subunits e, f, and g. Two additional subunits, known previously as 6.8 PL (6.8 kDa proteolipid) and diabetes associated protein in insulin sensitive tissue are referred to here according to the names of their yeast orthologs, subunits j and k, respectively (4, 5, 7–10). Sixteen of the 18 kinds of subunits of human ATP synthase are nuclear gene products that are imported into the organelle where they are assembled together to form the complete complex with the two remaining subunits ATP6 (or a) and ATP8 (or A6L), which are both encoded in mitochondrial DNA and synthesized in the mitochondrial matrix (11). Currently, it is not known when the five phospholipids are incorporated into the complex (*SI Appendix*, Fig. S1). Previously, we have described the later stages of the assembly of nuclear encoded subunits of the membrane domain based upon editing specific human genes with CRISPR-Cas9, by characterizing the partial vestigial ATP synthase complexes that assemble in these cells and by the study of the assembly process in ρ^0^ cells, which lack mitochondrial DNA and, therefore, are incapable of making subunits ATP6 and ATP8 (5, 12). From these studies an F_1_-PS-c_8_-ring complex has been identified as a fundamental assembly intermediate, that provides the template for insertion of the two mitochondrially encoded subunits ATP6 and ATP8 in an unknown order. Their insertion is completed by the addition of subunit j which augments the entry of ATP6, evidently by holding it in close juxtaposition to the c_8_ ring as required for the formation of the transmembrane proton pathway at the c_8_ ring:ATP6 interface. On the basis of the structures of the monomer:monomer interface in the dimeric bovine and yeast ATP synthases (4, 13), the entry of subunit j also allows the monomeric complexes to associate into the dimeric complexes found along the edges of the cristae in mitochondria (14–17). The final subunit to be added, subunit k may be involved in tethering dimers into tetramers and higher oligomers (5, 18). Here, we describe routes for assembly and incorporation of the PS module into the complex.

## Results

### Human Cells Lacking Subunits d and F_6_.

**Fig. 2.**
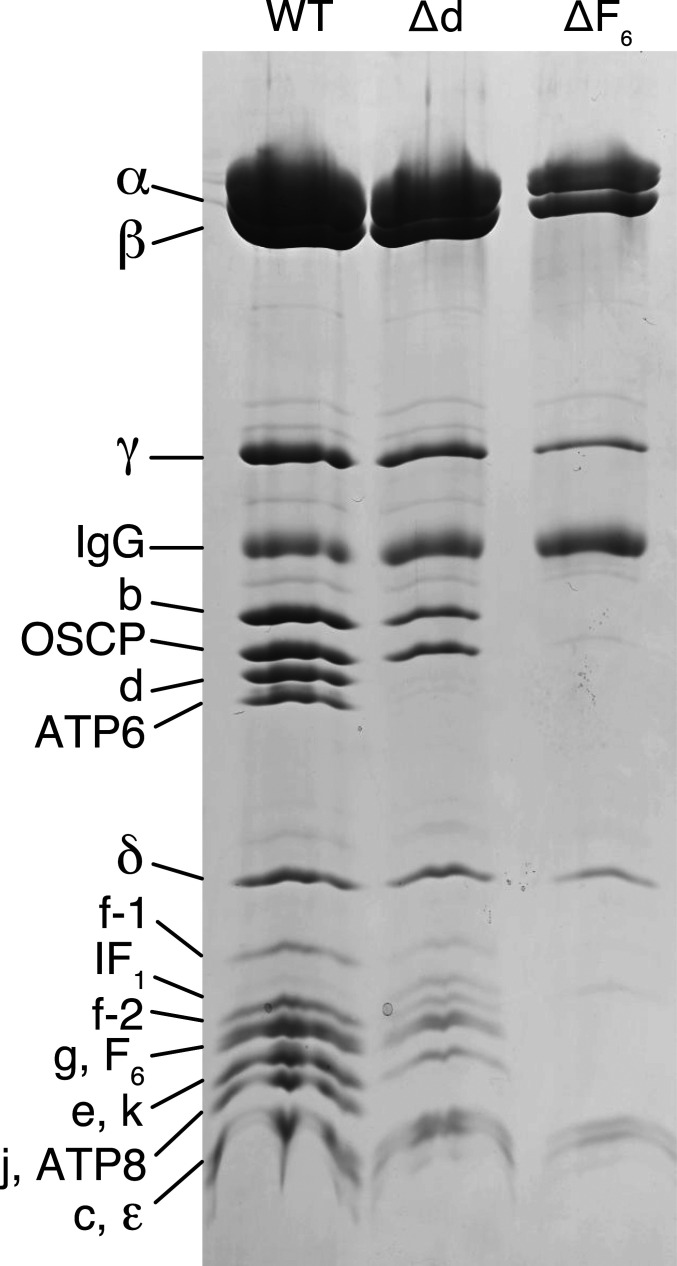
Vestigial ATP synthase complexes in HAP1 cells devoid of peripheral stalk subunits d and F_6_. Subunit compositions of vestigial human ATP synthase complexes in HAP1-∆d and HAP1-∆F_6_ cells. The vestigial complexes were immunopurified from digitonin extracts of mitoplasts, fractioned by SDS-PAGE, and proteins were detected with Coomassie blue dye. The positions of subunits are indicated on the left.

**Fig. 3.**
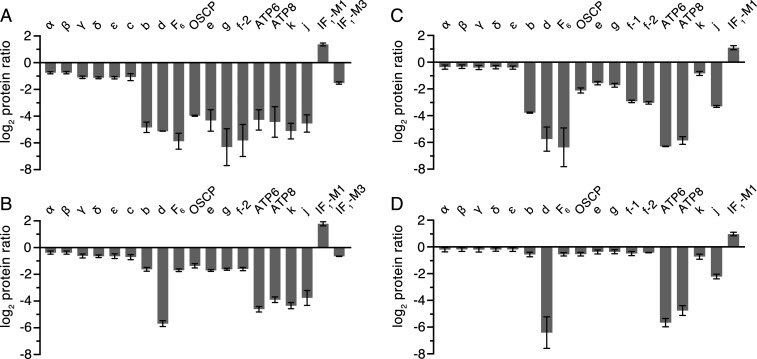
Impact of removal of either subunit F_6_ or d on the composition of vestigial ATP synthase complexes. Relative abundances of subunits and the inhibitor protein IF_1_ in HAP1-ΔF_6_ or HAP1-Δd cells, respectively, *A* and *B* in immunopurified vestigial complexes of ATP synthase or *C* and *D* in digitonin solubilized mitoplasts. IF_1_-M1 and IF_1_-M3 are specific mature forms of IF_1_ (5), and f-1 and f-2 are isoforms of subunit f (Swiss-Prot P56134). The digitonin extracts of mitoplasts were prepared from 1:1 mixtures of differentially SILAC-labeled HAP1-WT cells plus either HAP1-ΔF_6_ or HAP1-Δd cells, and complexes were purified from these samples. The purified samples were digested separately in solution with trypsin or chymotrypsin, and mitoplast samples were separated by SDS-PAGE and digested in gel with trypsin. Peptide mixtures were fractionated by reverse phase HPLC and analyzed by mass spectrometry. The histograms are the median values of both relative abundance ratios determined for proteins found in the complementary stable isotope labeling with amino acids in cell culture (SILAC) experiments. The protein ratio is derived from a minimum of two peptide ratios from each experiment except for ATP6 (*B* and *D*, control heavy isotope experiments), f-2 (*D*, both experiments), and IF_1_-M1 (*D*, control light isotope experiment) where all values are from a single peptide ratio. In mitoplast samples, no ratios were obtained for subunit c or IF_1_-M3 (*C* and *D*). The data for the identified proteins are given in Datasets S1–S8. Error bars show the range of the two values.

*ATP5PD* and *ATP5PF* encoding, respectively, subunits d and F_6_ of ATP synthase (*SI Appendix*, Fig. S2) were disrupted in HAP1-WT (wild-type) cells by CRISPR-Cas9 (*SI Appendix*, Tables S1 and S2 and Fig. S3). The resulting clonal cells, HAP1-Δd and HAP1-ΔF_6_, lacked the edited subunits and produced different vestigial complexes with PS subunits depleted or absent (Fig. 2 and *SI Appendix*, Fig. S4). The removal of either subunit d or F_6_ had little effect on the initial rate of cell proliferation, but after about 60–70 h, growth ceased and the confluence declined (*SI Appendix*, Fig. S5), probably because of increased glycolysis and faster acidification of the culture medium in mutant cells than in WT cells under noninhibited conditions (*SI Appendix*, Fig. S5). The deletion of subunit d or F_6_ affected respiration severely (*SI Appendix*, Fig. S5), similar to the impact of individual removal of the oligomycin sensitivity conferral protein (OSCP) or subunits b, c, e, f, and g (5, 12, 19). The lack of the effect of oligomycin on these cells indicated that any intermediate vestigial ATP synthases are uncoupled from pmf. The levels of complexes I, III, and IV, but not complex II, were lower in both mutant cells (*SI Appendix*, Fig. S6) with an associated lower respiratory capacity (*SI Appendix*, Fig. S5). The effect of removing the PS subunit F_6_ had a greater impact on assembly (Fig. 2), and the vestigial complex in HAP1-ΔF_6_ cells contained significant levels of only the five subunits from the F_1_ domain and the membrane subunit c (Fig. 3*A*, *SI Appendix*, Fig. S7*A*, and Datasets S1 and S2), consistent with the presence of a F_1_-c_8_ subcomplex, observed previously when either of two other PS constituent subunits, b or OSCP, was removed (19). The quantitative MS analysis (Fig. 3*A*) showed that the level of the vestigial F_1_-c_8_ complex in HAP1-ΔF_6_ cells was reduced to ca. 50% of the level of intact ATP synthase in HAP1-WT cells. Similarly, in HAP1-Δd cells, the constituent subunits of the F_1_-c_8_ component were present at ca. 60–65% of the levels in WT cells with the remaining PS and membrane subunits at ca. 30–40% of their WT abundances (Fig. 3*B*, *SI Appendix*, Fig. S7*B*, and Datasets S3 and S4). Therefore, it appears that HAP1-Δd cells assemble both a F_1_-c_8_ subcomplex and a F_1_-c_8_ subcomplex associated with a PS complex containing subunits OSCP, b, F_6_, and e, f, and g. An alternative interpretation is that the F_1_-c_8_-OSCP-F_6_-b-e-f-g vestigial complex in HAP1-Δd cells is somewhat unstable and dissociates partially during purification. The formation of the vestigial complexes in both derivative cells was accompanied by an elevation in the relative level of IF_1_-M1 and a decrease in IF_1_-M3 (Fig. 3 *A* and *B*).

The quantitative relative levels of subunits in mitoplasts showed that removal of subunit d was accompanied by a substantial reduction in the levels of mitochondrially encoded ATP synthase subunits ATP6 and ATP8, and the level of subunit j was reduced also (Fig. 3*D*, *SI Appendix*, Fig. S8*A*, and Datasets S5 and S6). None of these three subunits is a component of the vestigial complex in HAP1-Δd cells. The low levels of ATP6, ATP8, and subunit j in mitoplasts may reflect a rapid turnover of unassembled subunits or down-regulation of their expression in the absence of an assembled enzyme. A comparison of the relative levels of the other subunits in mitoplasts indicates a small excess of F_1_-c_8_ components, supporting the presence of both F_1_-c_8_ and F_1_-c_8_ associated with a PS complex containing subunits OSCP, b, F_6_, and e, f, and g. The deletion of subunit F_6_ was accompanied by a large decrease in the levels of subunits ATP6, ATP8, and d and low levels of b, OSCP, e, f, g, and j, consistent with these cells forming predominantly a F_1_-c_8_ vestigial complex (Fig. 3*C*, *SI Appendix*, Fig. S8*B*, and Datasets S7 and S8). In both mutant cells, the difference in the relative levels of subunit k in mitoplasts compared with the purified complexes was larger than for other ATP synthase subunits, indicating the presence of the free unassociated subunit.

### Oligomeric State of Vestigial ATP Synthases.

**Fig. 4.**
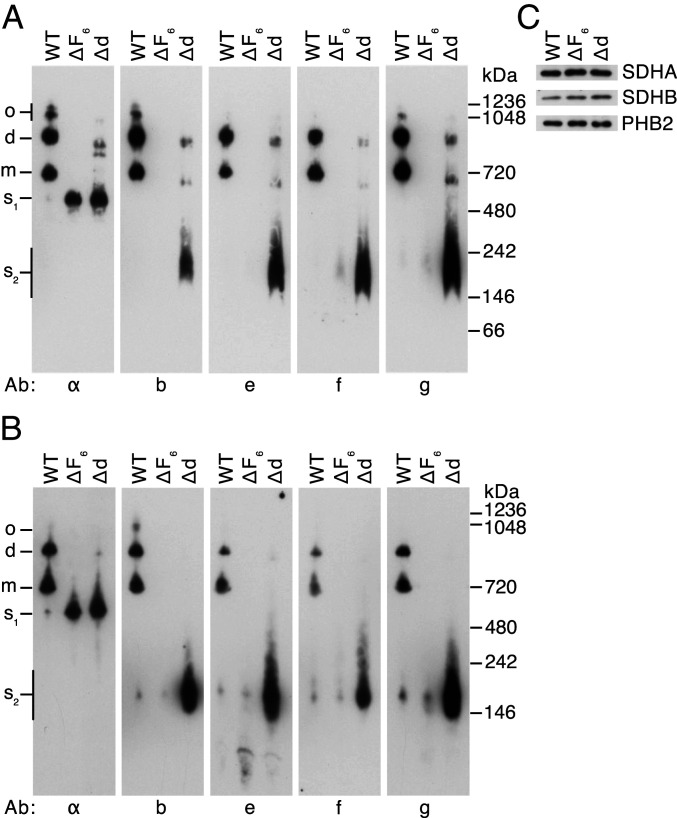
Oligomeric states of ATP synthase and vestigial complexes in HAP1 cells. Mitochondrial membranes from HAP1-WT, HAP1-ΔF_6,_ and HAP1-Δd cells were extracted with digitonin (6 g/g protein). (*A* and *B*) Fractionation of extracts by BN-PAGE and CN-PAGE, respectively. Complexes were revealed by Western blotting with antibodies against various subunits of ATP synthase (indicated below the panels). The positions of complexes are shown on the left: o, oligomers; d, dimers; m, monomers; s_1_, F_1_-c_8_ subcomplex; s_2_, subcomplexes containing subunits b, e, f, and g. The positions of molecular weight markers are shown on the right. (*C*), assessment of sample loading by Western blotting of digitonin extracts, fractionated by SDS/PAGE with antibodies against the succinate dehydrogenase A (SDHA) and SDHB subunits of complex II and prohibitin 2 (PHB2).

Analysis by both blue native polyacrylamide gel electrophoresis (BN-PAGE) and clear native polyacrylamide gel electrophoresis (CN-PAGE) of the vestigial ATP synthase from HAP1-ΔF_6_ cells demonstrated the presence of a single dominant band, cross reacting with an antibody for the α-subunit at a position corresponding to a monomeric F_1_-c_8_ subcomplex of ATP synthase (s_1_ in Fig. 4), supporting the quantitative analysis (Fig. 3*A*). A minor component at ca.160 kDa detected in CN-PAGE analysis contained, at least, the PS subunit b and supernumerary subunits e, f, and g (s_2_ in Fig. 4*B*), and a similar complex was detected in WT cells. This b-e-g-f containing subcomplex was prominent in analyses by both BN- and CN-PAGE of samples from HAP1-Δd cells. These latter samples also contained the F_1_-c_8_ subcomplex (s_1_), and additional bands detected in BN-PAGE gels could be monomeric and dimeric forms of a vestigial F_1_-c_8_-OSCP-F_6_-b-e-f-g complex (Fig. 4*A*). As subunit j, the key dimerizing component (4) is largely absent, the presence of dimers can be explained either by the face-to-back association of monomers via subunit g or by monomeric complexes linked as in the structure of tetrameric porcine ATP synthase (20) by dimeric IF_1_ (21, 22). The IF_1_-M1 form is consistently found at increased relative levels in the vestigial complexes (Fig. 3) (5, 12, 19). The quantitative mass spectrometric analyses suggested that HAP1-Δd cells produce both a F_1_-c_8_ and a F_1_-c_8_-OSCP-F_6_-b-e-f-g vestigial complex. The low intensity of the apparent monomeric and dimeric F_1_-c_8_-OSCP-F_6_-b-e-f-g vestigial complexes, combined with the strong b-e-f–g band suggests that the F_1_-c_8_-OSCP-F_6_-b-e–g vestigial complex may be unstable and give rise to the combination of complexes observed in the native gel analyses of samples from HAP1-Δd cells.

### The b-e-g and b-e-g-f Subcomplexes of ATP Synthase.

**Fig. 5.**
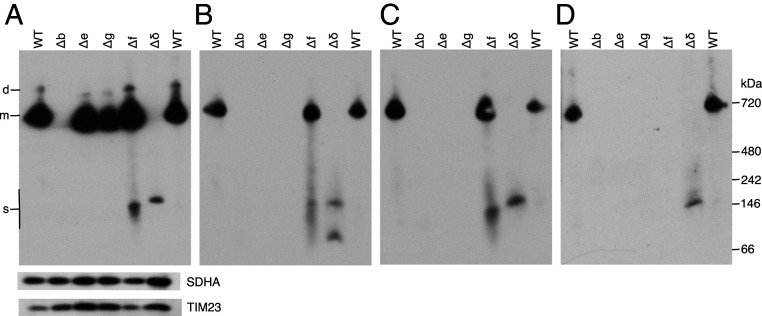
Oligomeric states of ATP synthase and vestigial forms in HAP1-WT, HAP1-∆b, HAP1-∆e, HAP1-∆g, HAP1-∆f, and HAP1-∆δ cells. ATP synthase and vestigial complexes were extracted from mitoplasts with digitonin (12 g/g protein). Extracts were fractioned by CN-PAGE, and complexes detected with antibodies against individual subunits of ATP synthase. (*A*), subunit b. (*B*), subunit e. The lower band in the ∆δ lane is probably free subunit e. (*C*), subunit g. (*D*), subunit f. The SDHA subunit of complex II and TIM23 are loading controls. On the left, d, and m, dimeric, and monomeric ATP synthase, respectively; s, subcomplex b-e-g-f in Δδ samples and b-e-g in HAP1-∆f samples. The positions of protein markers are shown on the right.

The formation of a b-e-g-f subcomplex in HAP1 cells was examined further in cell lines lacking individual subunits b, e, f, g, and δ. It was demonstrated that the HAP1-Δe, HAP1-Δg, and HAP1-Δf cells all form monomeric vestigial complexes containing the b subunit, slightly smaller than intact monomeric ATP synthase, and that the complex formed by HAP1-Δf cells also contains subunits e and g (Fig. 5) as observed before by BN-PAGE (5). Also, HAP1-Δf cells produced a smaller b-e-g subcomplex of ca. 150 kDa, and this subcomplex was not formed when any one of subunits b, e, or g was deleted. A monomeric vestigial complex of ca.700 kDa was not observed in HAP1-Δδ cells, but they have a subcomplex of ca.160 kDa, containing subunits b, e, g, and f, slightly larger than the b-e-g subcomplex as expected, and consistent with the assembly of a b-e-g-f subcomplex. The HAP1-Δδ cells also formed an uncharacterized 80 kDa complex containing subunit e.

**Fig. 6.**
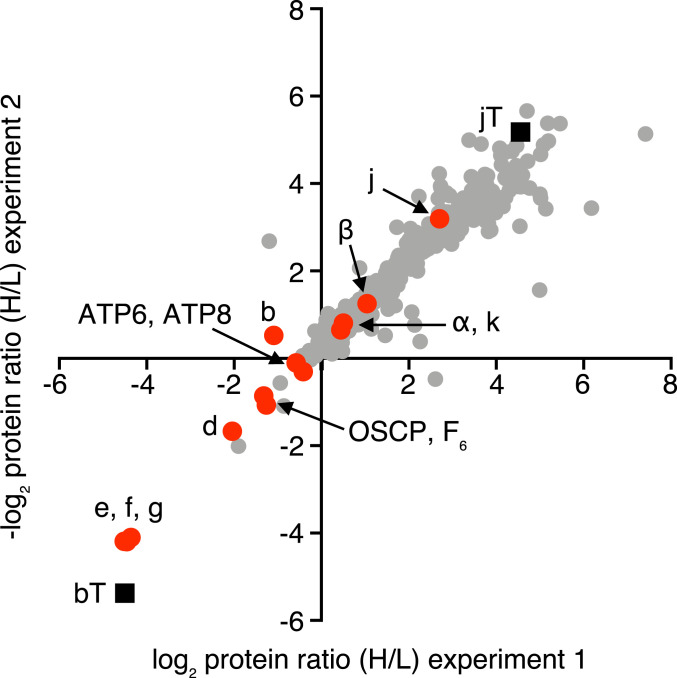
Proteins associated with subunits b and j in HEK293T-Δδ cells. Quantitative mass spectrometry of affinity purified FLAG-Strep II tagged subunit b (bT) and subunit j (jT) and associated proteins. SILAC-labeled HEK293T-Δδ Flp-In T-REx cells individually overexpressing the tagged proteins were grown separately, and combined and mitoplast samples prepared by a mild digitonin treatment. Proteins in mitoplast samples were extracted using digitonin at a higher detergent:protein ratio of 12:1 (g/g), and tagged plus associated proteins were purified by Strep II affinity chromatography. The experiment was performed twice with reciprocal SILAC labeling orientations. ■, tagged proteins. 
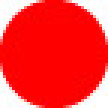
, ATP synthase subunits. 
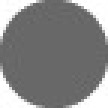
, Other proteins. Each data point corresponds to the abundance ratio of an identified protein from the two complementary experiments. The data for all of the identified proteins are given in Datasets S9–S11.

Further evidence for the b-e-g-f complex was provided by the expression in human embryonic kidney 293 (HEK293)-Δδ cells of the b subunit with a C-terminal FLAG-Strep II tag, and analysis of the purified complex of the tagged subunit and associated proteins by gel and mass spectrometric analyses. The HEK293-Δδ cell line was employed so as to favor the formation of the b-e-g-f subcomplex (*SI Appendix*, Fig. S9*A*) as in HAP1-Δδ cells (Fig. 5). The reciprocally labeled control sample for this experiment was provided by HEK293-Δδ cells where a C-terminally tagged version of the subunit j (which is not part of the b-e-g-f complex) was expressed. There was no evidence for or against a specific complex containing subunit j in the HEK293-Δδ cells (Fig. 6), but, as the majority of the data points occupy the upper right quadrant, the choice of tagged subunit j as a control was suboptimal. However, only subunits e, f, and g were appreciably associated with the tagged b subunit (Fig. 6), and, therefore, the experiment provides further evidence for the formation of the b-e-g-f intermediate.

## Discussion

The bovine and human ATP synthases have the same subunit composition, and the sequences of the subunits are highly conserved (3, 12, 23). Therefore, the structure of the bovine enzyme (Fig. 1) is an excellent surrogate for that of the human enzyme that can be employed in the interpretation of the pathway of assembly of the human complex. The structure illustrates that the protein components of the PS, the OSCP, b, d, F_6_, e, f, and g subunits, plus the ATP6 and ATP8 subunits, and the associated supernumerary membrane subunits j and k, constitute the enzyme’s stator against which the rotor turns (4). The rotor consists of central stalk components, subunits γ, δ, and ε, which is bound to a ring of eight c subunits in the membrane domain (24). During catalysis, the PS prevents the dissociation of the α_3_β_3_-domain from the central stalk by clamping it in position from above via interactions between the N-terminal regions of α-subunits with the N-terminal domain of the OSCP. Also, it resists the rotational torque of the central stalk, preventing the α_3_β_3_-domain and the rest of the stator domain from following the direction of rotation. The structure of the PS is elongated and largely α-helical, and it illustrates the roles of its individual components. The N-terminal domain of the OSCP, attached to the F_1_ domain, is linked to its C-terminal domain via a flexible region that provides a universal joint to accommodate the side-to-side rocking of the F_1_ domain during catalysis. This C-terminal domain of the OSCP is bound to bH4, the C-terminal α-helix of subunit b. α-Helix bH4 is attached to α-helix bH3, which extends about 150 Å through the core of the PS to the surface of the IMM and then across the membrane. Other α-helices in the F_6_ and d subunits and another extending up from the membrane domain of subunit ATP8 are bound parallel to bH3, helping to rigidify most of the PS, except for a hinge in bH3 close to the membrane’s surface that allows the PS to flex during catalysis. The membrane bound N-terminal region of the b subunit is folded into two further α-helices bH1 and bH2, and together with the transmembrane domain of bH3, they form the skeleton of a wedge-shaped structure with bH1 sitting on top on the matrix side of the IMM and transmembrane α-helices bH2 and bH3 subtending an angle of ca. 45° (*SI Appendix*, Fig. S1). The association of the two wedges in the dimeric enzyme places the central rotatory axes of the monomeric enzymes at an angle to each other. The supernumerary subunits e, f, and g also contribute to the structure of the wedge. The single transmembrane α-helices of each of subunits e and g augment α-helix bH2 and the top of the wedge on the matrix side of the membrane is provided by four amphipathic α-helices two in each of the g and f subunits, which lie in the lipid head-group region of the membrane. Internal cavities in the wedge are occupied by five specifically bound lipids, three cardiolipins (CDL1–CDL3) and two other lipids tentatively modeled as phosphatidyl glycerols (LHG4 and LHG5). These five lipids probably enhance the stability of the wedge (4).

**Fig. 7.**
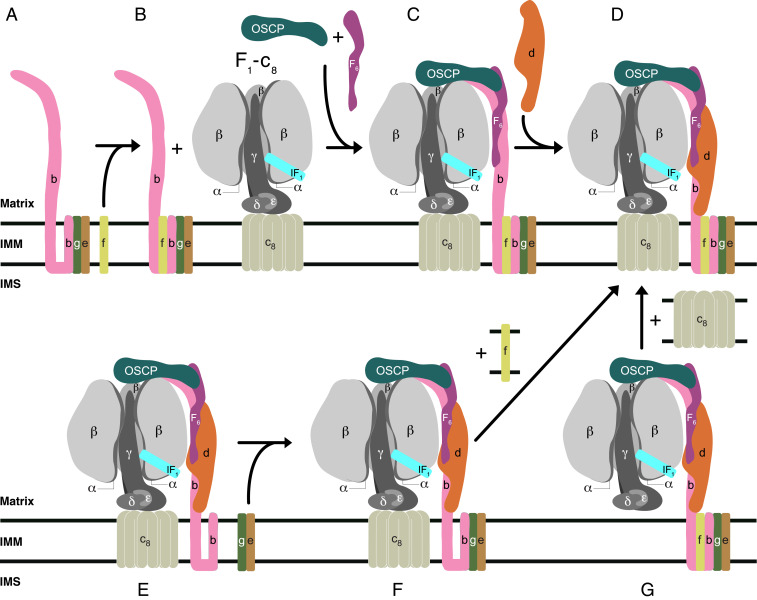
Pathway(s) of the assembly of the PS of human ATP synthase. (*A* and *B*) A tertiary b-e-g complex observed in HAP1-Δf cells binds subunit f to form the complex observed in HAP1-Δδ cells; *C* in the presence of the OSCP and F_6_, the b-e-g-f subcomplex binds to the F_1_-c_8_, complex to form the partially stable F_1_-c_8_-OSCP-F_6_-b-e-g-f subcomplex observed in HAP1-Δd cells. (*D*) The stable key intermediate vestigial complex observed in ρ^0^ cells is formed by the addition of the d subunit; (*E, F,* and *G*), two additional pathways of arriving at intermediate (*D*); in *E*, a partly stable vestigial complex observed in HAP1-Δe and HAP1-Δg cells is elaborated by the addition of subunits e and g to form complex (*F*) observed in HAP1-Δf cells; alternatively, key intermediate (*D*) is formed from (*G*), observed in HAP1-Δc cells, and the independently assembled c_8_ ring. Black horizontal lines represent the IMM separating the matrix from the IMS.

In earlier studies of the assembly of the membrane domain of human ATP synthase, subcomplex (*D*) in Fig. 7 was characterized as a key intermediate in the later stages of the assembly process (5). It provides the template for the introduction of subunits ATP6 and ATP8, both encoded in mitochondrial DNA, allowing the proton channel to be formed and then stabilized by the subsequent addition of subunit j, producing a coupled active ATP synthase. In subcomplex (*D*), the PS is already fully assembled. In the current paper, other vestigial complexes have been characterized from the mitochondria of human cells where various subunits of ATP synthase have been removed individually. They demonstrate that the PS is not introduced into intermediate (*D*) by a unique route, but that there are, at least, three alternative paths for doing so (Fig. 7). In one path, illustrated in Fig. 7 *A* and *B*, a b-e-g complex binds subunit f to form a b-e-g-f subcomplex. When the association of this assembly with F_1_-c_8_ was prevented, for example, in HAP1-ΔF_6_ cells, then b-e-g-f did not accumulate (Fig. 4), presumably because it was degraded in the mitochondria. The b-e-g-f subcomplex is probably an authentic assembly intermediate as it forms in the absence of an assembled F_1_ domain (Fig. 6), but it cannot be completely excluded that it is also the product of the breakdown of an F_1_-c_8_-PS subcomplex, for example, either metabolically or artifactually during extraction of the vestigial complex with detergent and subsequent gel analyses. For example, in HAP1-Δd cells (Fig. 4), the substantial amounts of b-e-g-f that were observed are likely to have arisen from partial dissociation of an unstable F_1_-c_8_-OSCP-F_6_-b-e-f-g vestigial complex. A b-e-g complex, that probably contained subunit f also, has been described before in HeLa cells where subunit d had been depleted (25). According to Fig. 7, the b-e-g-f subcomplex then associates with the F_1_-c_8_ subcomplex in the presence of subunits OSCP and F_6_, resulting in the formation of the substoichiometric intermediate (*C*). In a final step, the assembly of the PS is completed by the introduction of subunit d. In a second path to intermediate (*D*) in Fig. 7 *E* and *F*, a F_1_-c_8_ subcomplex, already associated with a partial PS consisting of subunits OSCP, b, d, and F_6_, binds an e-g subcomplex, and then intermediate (*D*) is made by the addition of subunit f. In a third path, the intermediate (*D*) is formed by first the association of a F_1_ domain with a fully assembled PS and then completed by the addition of the c_8_ ring. A fourth, but related pathway of assembly of human ATP synthase has been proposed based on the mass spectrometric analysis of protein complexes fractionated by native gel electrophoresis (26). Here, a b-e-g-f subcomplex becomes incorporated into a PS complex plus subunits ATP6, ATP8, j, and k, which then associates with a F_1_-c_8_ subcomplex to produce the intact ATP synthase. From current knowledge, it is not possible to say which route, if any, is dominant, and under what circumstances the alternative routes operate. In *Saccharomyces cerevisiae*, subunit c as well as ATP6 and ATP8 are encoded in the organellar genome. Here, a F_1_-PS-ATP6-ATP8 subcomplex forms first and then the c_10_ ring is incorporated (6). However, the formation of the intermediate subcomplex requires the participation of assembly factors Atp10, Atp23, and INAC (27). So far, no assembly factor has been found to be required for the assembly of the human PS (Fig. 6 and *SI Appendix*, Fig. S7), although others are needed to assist in the assembly of the F_1_ domain (28, 29) and creating the c_8_ ring and/or its association with the F_1_ domain (30).

The formation of a b-e-g complex followed by the addition of subunit f to make the b-e-g-f subcomplex is consistent with the structure of the wedge domain (4) since subunit f is bound partially to the external surface of subunit g (*SI Appendix*, Fig. S1). From consideration of the structure, it seems likely that phospholipids CDL2, LHG4, CDL3, and LHG5 will be added to the b-e-g complex (effectively a nascent wedge complex) at as yet unspecified points before the addition of subunit f and that CDL1 will be added subsequently (*SI Appendix*, Fig. S1). The incorporation of these lipids into the wedge domain of the ATP synthase is part of the assembly process of the enzyme, and it warrants further investigation. Another issue requiring clarification is at what stage of the assembly process do the monomeric complexes dimerize? Two types of dimer can form, made from either appropriate vestigial complexes or from the fully assembled monomeric complex. They are the classic front-to-back dimers held together by interactions between j subunits (4) with the rotatory axes at a range of acute angles and those arising by the association of two monomers (partially or fully assembled) across what will become the dimer–dimer interface in the rows of dimers observed in the cristae. The latter class could arise from interactions between k subunits in each monomer (5), or they could be held together by dimeric IF_1_ molecules bridging between the two catalytic F_1_ domains (20, 21). Low levels of dimeric forms of vestigial ATP synthase complexes lacking subunit j have been observed in ρ^0^, Δd, and Δf cells (Fig. 4) (5, 12, 31), suggesting that they may be side-by-side dimers. Conditions are known for extracting and purifying dimeric bovine complexes that have been verified by electron cryomicroscopy to be front-to-back dimers (4). Dimeric complexes are frequently observed in native gel analyses of extracts of mitochondrial membranes from WT and mutant cells and are often assumed to be the classic type. However, the exact nature of the dimer cannot be ascertained with certainty without corroborative investigations, and improved protocols are required for the study of their involvement in the assembly of the ATP synthase.

## Materials and Methods

Human genes *ATP5PD* and *ATP5PF* in HAP1 cells and *ATP5F1D* in HEK293 Flp-In T-REx cells were disrupted by clustered regularly interspaced short palindromic repeats-Cas9 (32), leading to clonal cells HAP1-Δd, HAP1-ΔF_6_, and HEK293-Δδ, respectively. HAP1-WT and a clonal HAP1-Δδ cell were purchased from Horizon Discovery, Cambridge, UK. The derivation of HAP1-∆b, HAP1-∆e, HAP1-∆f, and HAP1-∆g cells has been described before (5, 19). Plasmid pcDNA5/FRT/TO encoding either subunit b or j, each with tandem C-terminal Strep II and FLAG tags was cotransfected with plasmid pOG44 in the presence of Lipofectamine 2000 (Invitrogen) into HEK293-Δδ cells, and stably transformed cells expressing the tagged subunits were selected. Cell proliferation was monitored with an Incucyte HD instrument (Essen Bioscience), and the oxygen consumption of cells was measured in a Seahorse XF^e^24 analyzer (Agilent Technologies). The o state of ATP synthase and vestigial complexes was examined by BN-, CN-PAGE, and Western blotting. ATP synthase and vestigial complexes were immunopurified from mitoplasts, analyzed by SDS/PAGE with Coomassie blue staining and by quantitative mass spectrometry of trypsin and chymotrypsin digests. For quantitative MS analyses, proteins were subject to SILAC. For details, see the *SI Appendix*.

## Supplementary Material

Supplementary File

Supplementary File

## Data Availability

All study data are included in the article and supporting information.
