# Supplementary material for: Assembly of the peripheral stalk of ATP synthase in human mitochondria
Source: Proc Natl Acad Sci U S A. 2020 Nov 9;117(47):29602–8. doi: 10.1073/pnas.2017987117 (PMC7703580; doi:10.1073/pnas.2017987117)
Supplement: Supplementary File [file pnas.2017987117.sapp.pdf]

PNAS
www.pnas.org

Supplementary Information for

Assembly of the peripheral stalk of ATP synthase in human mitochondria

Jiuya He, Joe Carroll, Shujing Ding, Ian M. Fearnley, Martin G. Montgomery and John E. Walker
Medical Research Council Mitochondrial Biology Unit, University of Cambridge, Cambridge Biomedical Campus, Hills Road, Cambridge CB2 0XY, United Kingdom

Corresponding author: John E. Walker
Email: walker@mrc-mbu.cam.ac.uk

This PDF file includes:

Supplementary text
Figures S1 to S10
Tables S1 to S3
Legends for Datasets S1 to S11
SI References

Other supplementary materials for this manuscript include the following:

Datasets S1 to S11

Supplementary Materials and Methods

Gene Disruptions. *ATP5PD* and *ATP5PF* encoding the d and F_6 subunits were disrupted individually in human HAP1-WT cells by CRISPR-Cas9 technology (1). Their structures are shown in *SI Appendix*, Fig. S2. HAP1 cells have a haploid karyotype, except for the presence of a fragment of chromosome 15 in chromosome 19 and a reciprocal translocation between chromosomes 9 and 22 (2, 3). None of these features affects the genes in question, as *ATP5PD* and *ATP5PF* are on chromosomes 17 and 21, respectively. For *ATP5PD* a pair of gRNAs characteristic of exon II and intron B were selected, and for *ATP5PF* the gRNA pair were both targeted to exon II (*SI Appendix*, Table S1). Each pair was introduced independently into HAP1-WT cells, and screening of clones from single cells for the absence of subunit d or F_6 led to the identification of HAP1-Δd, and -ΔF_6 cells. The changes introduced into *ATP5PD* and *ATP5PF* were sequenced with specific primers (*SI Appendix*, Table S2), showing that deletions had been introduced of 121 and 93 bp, respectively (*SI Appendix*, Fig. S3). Each deletion had arisen from two gRNAs and was mediated by non-homologous end-joining of the deleted genomic DNA. The deletion in *ATP5PD* left only the translational start codon in exon II, and also removed two bases of intron B, resulting in the termination of translation after 6 amino acids. An in-frame deletion in exon II of *ATP5PF* removed codons for amino acids 13-43, encompassing 20 residues of the mitochondrial targeting sequence and 11 amino acids at the N-terminus of the mature protein sequence. There was no evidence of the targeted protein in mitoplast samples from these cells (*SI Appendix*, Fig. S4). The HAP1-Δδ cells were purchased from Horizon Discovery (Cambridge, U. K.) who had used the single gRNA GCTGGTCGTGGTGCATGCA to disrupt the gene. The targeted region of the *ATP5F1D* gene was amplified by PCR with the forward and reverse primers 5'-GTGCCTCACATCAGCGCCAGGTC-3' and 5'-AGCAGGGTCCCCTCTGGTTCGC-3', respectively. This G-C rich region was sequenced by dGTP chemistry (Source Bioscience) showing that the mutation had been introduced into the same location as described previously in a HAP1-Δ(c+δ) cell line (4). The production and characterization of HAP1-Δb, -Δe, -Δf, and -Δg cells have been described previously (5, 6).

Human *ATP5F1D*, encoding the δ-subunit of ATP synthase was disrupted in a HEK293 Flp-In™ T-REx™ cell by CRISPR-Cas9 with the same gRNA used for HAP1 cells. The targeted region was amplified by PCR with the same primers that were used in HAP1 cells. The two resulting fragments observed by agarose gel electrophoresis were gel purified (QIAquick gel extraction kit, Qiagen), cloned into the pCR4-TOPO vector (Thermo Scientific), and sequenced with M13 forward and reverse primers, and the dGTP chemistry method (Source Bioscience). The HEK293-Δδ clonal cell is unable to produce a functional δ-subunit (Fig. S9 and S10). An aberrant 17-18 kDa protein of unknown sequence observed in SDS lysates of cells and mitoplasts of HEK293-Δδ cells cross-reacted with an anti-δ antibody, but it was unable to promote assembly of intact ATP synthase, as shown by native gel analysis (Fig. S9).

Cell Culture. HAP1-WT (Horizon Discovery) and clonal cells were cultured in Iscove's modified Dulbecco's medium under standard conditions (7). Cell proliferation was monitored with an Incucyte HD instrument (Essen Bioscience) and oxygen consumption rate (OCR) was measured in a Seahorse XF^{e}24 analyzer (Agilent Technologies), as described before (7). OCR was normalized to cell number by the sulforhodamine B assay (8). Stable isotope labelling of proteins with amino acids in cell culture (SILAC) (9) of HAP1-WT and gene disrupted clonal cells was carried out as described before (7).

Expression and Affinity Purification of Tagged ATP Synthase Subunits. Plasmid pcDNA5™/FRT/TO encoding subunit b or j, with tandem C-terminal Strep II and FLAG tags (10) was co-transfected in the presence of Lipofectamine 2000 (Invitrogen) with plasmid pOG44 into human HEK293-Δδ Flp-In™T-REx™ cells (total DNA, 1 µg; pOG44:pcDNA5/FRT/TO, 7:1 by weight). After 24 h, the medium was replaced with the selective medium containing blasticidin (10 µg/ml) and hygromycin (100 µg/ml) and inducible cell clones expressing the recombinant protein were picked and verified by Western blotting of cell lysates with an anti-Strep II antibody. SILAC-labelling of cell proteins in culture was performed as described before (11), and expression of tagged subunits was induced for 72 h with doxycycline (20 ng/ml) prior to harvesting the cells. The

SILAC DMEM media was also supplemented with 20 mM HEPES (pH 7.4) to aid buffering of the increased acidification of the media by these cell clones. Reciprocally labelled HEK293T-Δδ-bT (tagged subunit-b) and HEK293T-Δδ-jT (tagged j subunit) were mixed 1:1 (protein w/w). Mitoplast material was prepared by digitonin treatment (11, 12) and then solubilized with digitonin (12 g/g protein) for affinity purification of tagged subunits and associated proteins. A sample was loaded at 4°C onto a Pierce spin column (0.9 ml; Thermo Fisher Scientific) containing Strep-Tactin-Sepharose (IBA Lifesciences) and then washed with 5 column volumes of buffer [20 mM HEPES, pH 7.6, 150 mM NaCl, 2 mM dithiothreitol, 1x cOmplete EDTA-free protease inhibitor (Roche), and 0.05% (w/v) digitonin]. Bound protein was eluted with 6 portions of 0.5 column volumes of buffer containing 10 mM desthiobiotin. Eluates were analyzed by SDS-PAGE and quantitative mass spectrometry. A distinction was made between the endogenous subunits and the tagged form by the different migration positions on the SDS-PAGE gel. From the MaxQuant evidence file for the appropriate gel sections, the protein ratios of the tagged and endogenous subunits were determined manually (*SI appendix*, Datasets S10 and S11).

General Methods. Cell protein concentrations were determined by either the bicinchoninic acid assay (Thermo Fisher Scientific) or the detergent compatible protein assay (BioRad). Mitoplasts were prepared from cells with digitonin, as described before (11, 12). Extracts of mitoplasts made with dodecylmaltoside (DDM; 1%, w/v) were fractionated by SDS-PAGE, and subunits of ATP synthase and citrate synthase were detected by Western blotting. The oligomeric states of ATP synthase and vestigial complexes in digitonin extracts of mitoplasts were examined by BN-PAGE or CN-PAGE (13, 14), and Western blotting. Samples of mitoplasts were re-suspended to *ca.* 5 mg/ml in NativePAGE sample buffer (Thermo Fisher Scientific) containing digitonin (6-12 g/g protein), kept at 4°C for 15 min, and then centrifuged (10,500 x *g*, 20 min, 4°C). The supernatants were treated with benzonase (Merck Millipore) at room temperature, centrifuged again, and soluble complexes fractionated at 4°C in 3-12% acrylamide gradient Bis-Tris gels (Thermo Fisher Scientific) by CN-PAGE, or BN-PAGE according to the manufacturer's instructions for Western blotting. For CN-PAGE the cathode running buffer contained 0.05% (w/v) sodium deoxycholate plus 0.005% (w/v) DDM. The gel resolved complexes were transferred to polyvinylidene fluoride membranes, and the membranes were probed with subunit specific antibodies. The origins of the antibodies either have been described before (5, 6), or they are listed in *SI Appendix* Table S3. ATP synthase was purified from digitonin solubilized mitoplasts with an immuno-capture resin (Abcam) as described before (7). Proteins in SILAC labelled mitoplast samples for quantitative mass spectrometric analysis were reduced and alkylated in gel sample buffer, fractionated by SDS-PAGE and stained with Coomassie blue R250 dye (15). Stained gel sections were excised and proteins digested in-gel with trypsin (16). SILAC labelled and affinity purified samples of ATP synthase were ethanol precipitated at -20ºC for 18 h with 20 vol. cold ethanol, centrifuged, and the pellet was digested in 50 mM ammonium bicarbonate for 18 h, with either trypsin at 37ºC or chymotrypsin at 30ºC.

Protein Quantitation. Relative quantitation of proteins was derived from mass spectrometric data of SILAC samples (9). Peptide mixtures were analyzed by LC-MS-MS on a Proxeon EASY-nLC1000 system coupled directly to a Q-Exactive+ Orbitrap mass spectrometer (Thermo Fisher Scientific). Heavy and light peptide mass data were analyzed with MaxQuant version 1.6.5.0, and the integrated Andromeda search engine (17, 18) employing a Swiss-Prot human protein database (March 2019) modified to include mature forms of ATP5IF1, denoted as IF_1-M1, -M2 and -M3, with N-terminal residues Phe-25, Gly-26 and Ser-27, respectively (19, 20), and a mature ATP synthase c-subunit. The ATP synthase c-subunit is encoded by three genes (21, 22), with different mitochondrial targeting pre-sequences, but identical mature protein sequences. To aid identification of this subunit, using peptides derived from the mature protein N-terminal sequence, a representative sequence (lacking the mitochondrial N-terminal import sequence) was added to the human protein database employed in these analyses, with the identifier P48201-M. In addition, lysine trimethylation was included when interrogating chymotrypsin digest data, to aid identification of a characteristic methylated subunit-c peptide (23). Search parameters for in-gel trypsin digest samples were: MS tolerance 4.5 p.p.m.; MS/MS tolerance 20 p.p.m.; Trypsin/P with two missed cleavages; Fixed modification - Cys-carbamidomethyl; variable modifications - oxidation (Met) and

acetyl (protein N-terminus); Arg-10 and Lys-8. In solution digest sample parameters were: MS tolerance 4.5 p.p.m.; MS/MS tolerance 20 p.p.m.; trypsin/P with two missed cleavages; chymotrypsin with four missed cleavages; variable modifications - oxidation (Met), acetyl (protein N-terminus) and trimethyl (lysine); Arg-10 and Lys-8. The MaxQuant output was managed further with Perseus (24). Protein ratios for ATP5IF1 were calculated manually with data for the unique N-terminal peptides of the various mature forms of ATP5IF1, located in the MaxQuant evidence file, and represent the median of the assigned specific peptide ratios, where MaxQuant ISO-MSMS peptide values were used only if fewer than three MULTI-MSMS peptide ratios were obtained. The basis of quantitative experiments using SILAC has been described previously (25).

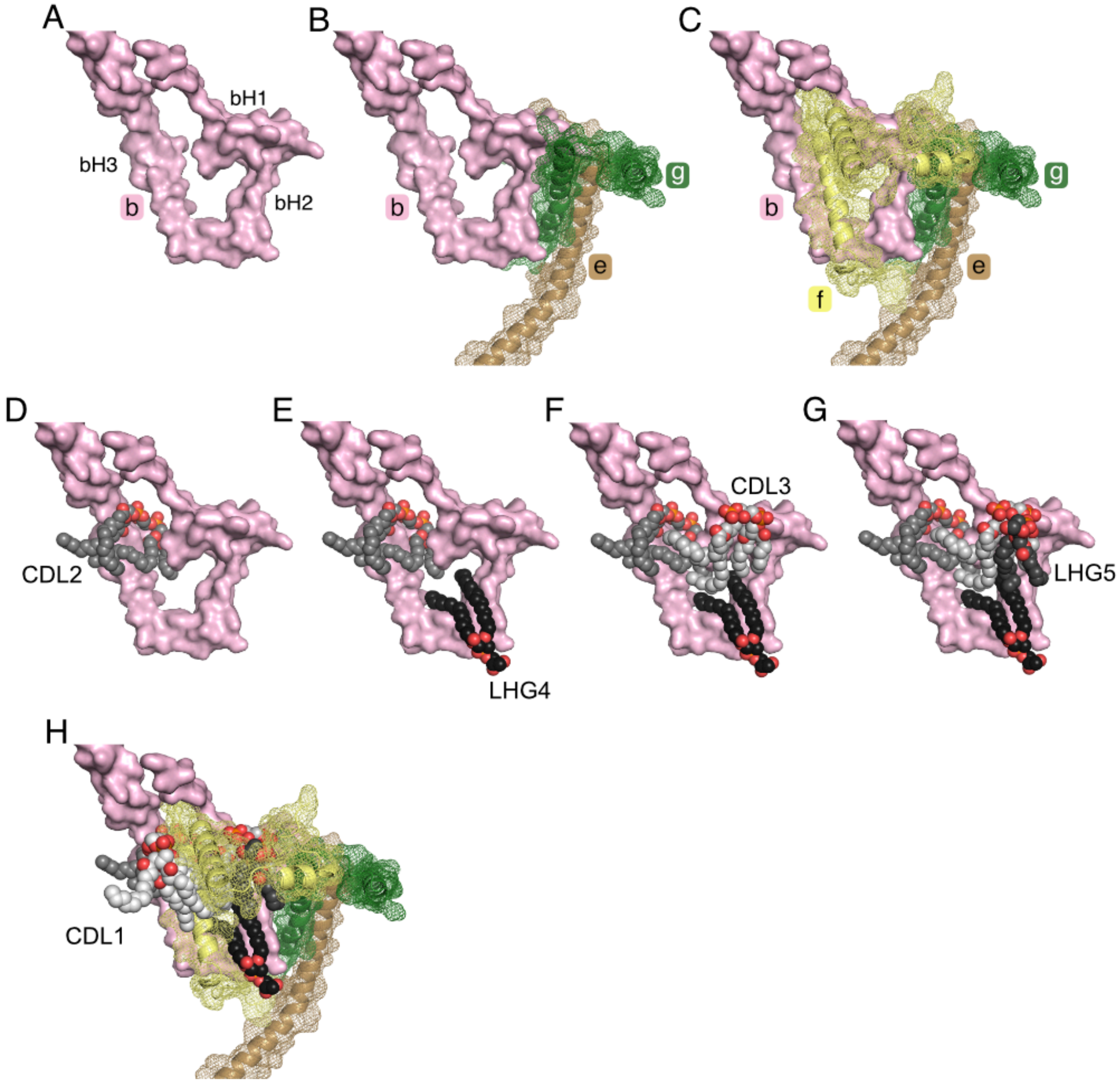

Fig. S1. Structure of wedge components in the membrane domain of bovine ATP synthase and possible points and order of incorporation of lipids into the wedge in the human enzyme. See (26). *A*, the membrane domain of subunit b, viewed orthogonal to the plane of the IMM, consisting of amphipathic α-helix bH1 (residues 19-29) in the head-group region on the matrix side of the IMM, transmembrane α-helix bH2 (residues 33-47) and the membrane region (residues 55-73) of bH3. In *B*, according to the assembly pathways in Fig. 7, subunits e and g associate with b to form the b-e-g intermediate. In *C*, subunit f is incorporated subsequently to complete the protein components of the wedge; *D-H*, possible points of incorporation of lipids. In *E-G*, CDL3, LGH4 and LGH5 could be incorporated before or after the addition of e and g to b to form b-e-g, but before incorporation of f. In *H,* CD1 is incorporated after the formation of the b-e-g intermediate to complete the b-e-g-f complex.

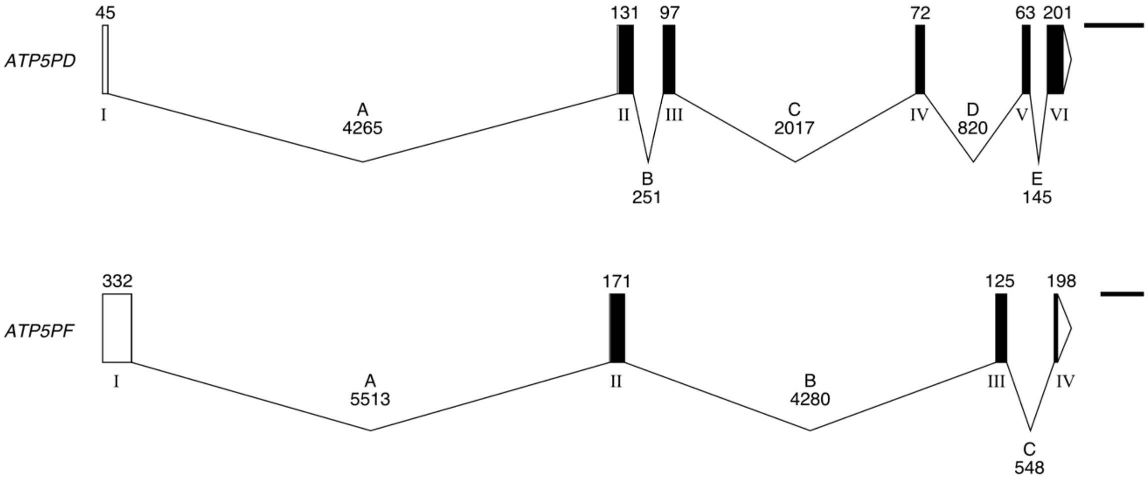

Fig. S2. Structures of the human genes *ATP5PD* and *ATP5PF* encoding subunits d and F_6, respectively, of ATP synthase. Exons are labelled with Roman numerals. Black and unfilled boxes in exons represent, respectively, protein coding and non-coding regions. Introns labelled with capital letters are depicted as intervening continuous lines. The sizes of introns and coding regions of exons are given in base pairs (bp). The scale bars on the right represent 500 bp. The exon-intron information was obtained from http://www.ensembl.org. The structures of *ATP5PD* and *ATP5PF* correspond to transcript ID reference ENST00000301587.9 and ENST00000400087.7. Images were drawn with the Exon-Intron graphic maker (http://wormweb.org/exonintron).

A

```
WT  GACTTATTTTTCAGGATCCCAAAATGGCTGGGCGAAAACTTGCTCTAAAAACCATTGACT
Δd  GACTTATTTTTCAGGATCCCAAAATG----------------------------------

WT  GGGTAGCTTTTGCAGAGATCATACCCCAGAACCAAAAGGCCATTGCTAGTTCCCTGAAAT
Δd  ------------------------------------------------------------

WT  CCTGGAATGAGACCCTCACCTCCAGGTCAGTATGTTTCTGAGAGGGAACCCCATACTTGC
Δd  ---------------------------CAGTATGTTTCTGAGAGGGAACCCCATACTTGC


WT  MAGRKLALKTIDWVAFAEIIPQNQKAIASSLKSWNETLTSRLAALPENPPAIDWAYYKANVAKAGLV
Δd  MVGCFT-------------------------------------------------------------
     *****

WT  DDFEKKFNALKVPVPEDKYTAQVDAEEKEDVKSCAEWVSLSKARIVEYEKEMEKMKNLIPFDQMTIE
Δd  -------------------------------------------------------------------

WT  DLNEAFPETKLDKKKYPYWPHQPIENL
Δd  ---------------------------
```

B

```
WT   TGTTTTCTGTAACAGAATCAGCATGATTCTTCAGAGGCTCTTCAGGTTCTCCTCTGTCAT
ΔF6  TGTTTTCTGTAACAGAATCAGCATGATTCTTCAGAGGCTCTTCAGGTTCTCCTCTG----

WT   TCGGTCAGCCGTCTCAGTCCATTTGCGGAGGAACATTGGTGTTACAGCAGTGGCATTTAA
ΔF6  ------------------------------------------------------------

WT   TAAGGAACTTGATCCTATACAGAAACTCTTTGTGGACAAGATTAGAGAATACAAATCTAA
ΔF6  -----------------------------TTGTGGACAAGATTAGAGAATACAAATCTAA

WT   GCGACAGTAAGTGAATAAATAAC
ΔF6  GCGACAGTAAGTGAATAAATAAC


WT   MILQRLFRFSSVIRSAVSVHLRRNIGVTAVAFNKELDPIQKLFVDKIREYKSKRQTSGGPVDASSEY
ΔF6  MILQRLFRFSSV-------------------------------VDKIREYKSKRQTSGGPVDASSEY

WT   QQELERELFKLKQMFGNADMNTFPTFKFEDPKFEVIEKPQA
ΔF6  QQELERELFKLKQMFGNADMNTFPTFKFEDPKFEVIEKPQA
```

Fig. S3. Sequences of genes *ATP5PD* and *ATP5PF* disrupted by CRISPR-Cas9, and the encoded protein sequences in clonal HAP1-Δd and -ΔF_6 cells. The sequences are compared to the corresponding wild-type (WT) sequences. (*A*) *ATP5PD* and d subunit and (*B*) *ATP5PF* and F_6 subunit. Carets indicate the PAM (protospacer adjacent motif) sequences for each guide RNA, and solid lines for the target sequences for guide RNAs. The WT sequences are parts of intron A (grey box), all of exon II and part of intron B (grey box), aligned with the corresponding deleted sequences. The arrows indicate the start codons in exon II. The deleted regions are denoted by dashed lines, and asterisks indicate non-matched amino acid to the wild-type.

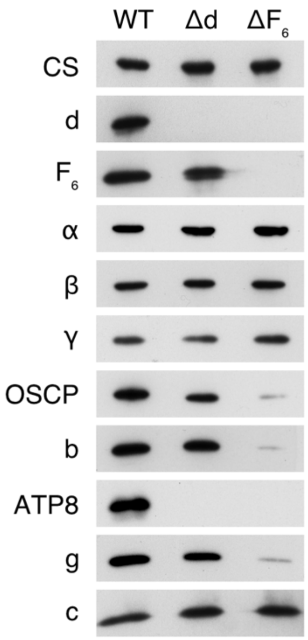

Fig. S4. Deletion of subunits d and F_6 from the peripheral stalk region of human ATP synthase in HAP1 cells. Mitoplasts from HAP1-WT, -Δd and -ΔF_6 clonal cells were extracted with *n*-dodecyl-β-D-maltoside. The extracts were fractionated by SDS-PAGE. Then the proteins were transferred to membranes and probed with antibodies against subunits indicated on the left. Citrate synthase (CS) was employed as a loading control.

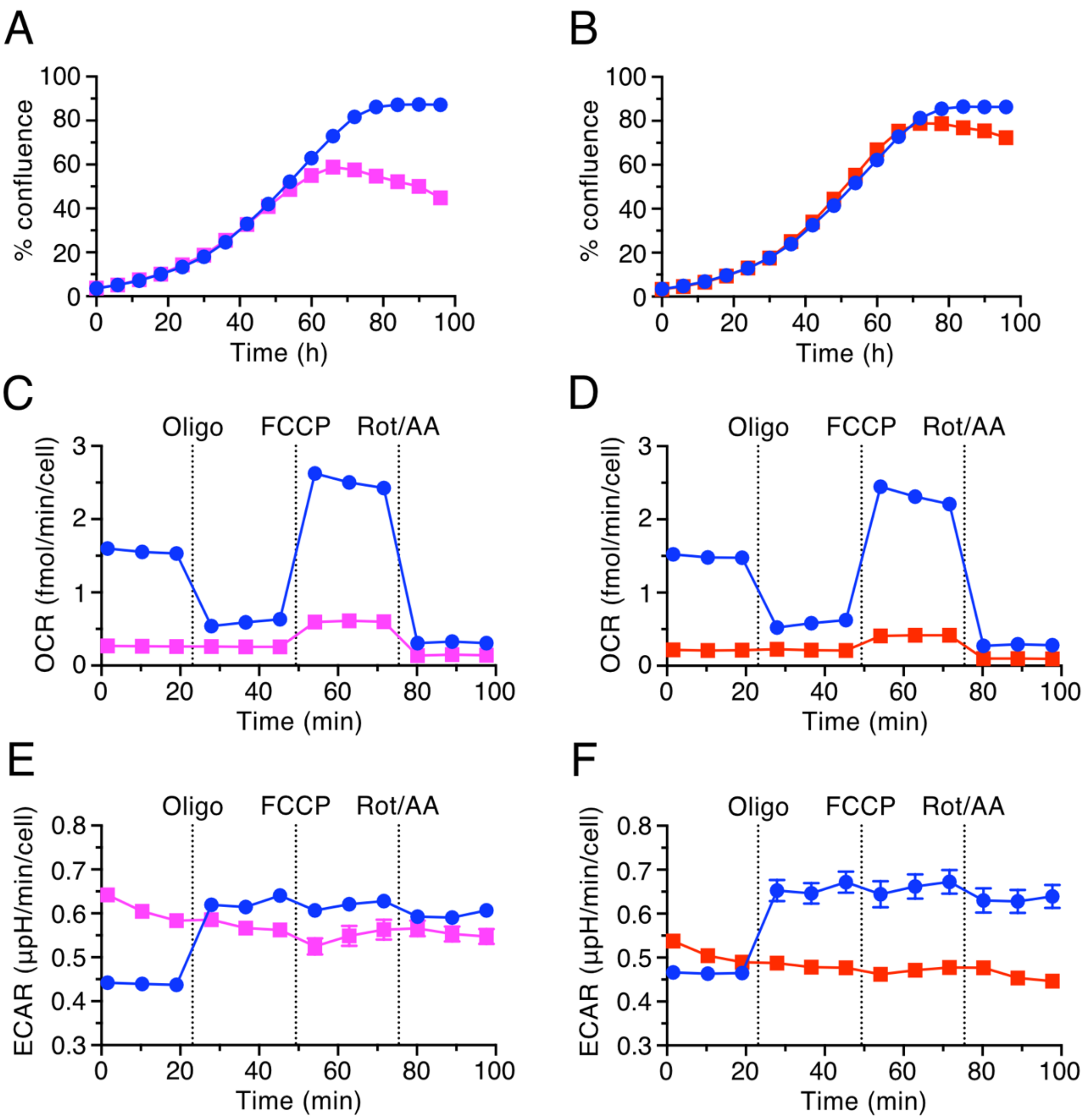

Fig. S5. Characteristics of HAP1-Δd cells and HAP1-ΔF_6 cells. (*A* and *B*), growth rates; (*C* and *D*) cellular oxygen consumption rates (OCR); (*E* and *F*) extracellular acidification rates (ECAR), of HAP1-WT cells (●) and HAP1 cells with disrupted genes for subunit d (■), or subunit F_6 (■). Growth rates were measured by seeding 10^5 cells into each well of a 6-well plate, and by monitoring their confluence over time. Initial confluences were adjusted to similar levels for comparison. The data points are the mean values ± SD (*n* = 3 wells). OCR and ECAR were measured with a Seahorse XF^e24 instrument, before and after sequential additions of oligomycin (Oligo), carbonyl cyanide-4-(trifluoromethoxy)phenylhydrazone (FCCP), and a mixture of rotenone and antimycin A (Rot/AA). Data represent the mean ± SEM (*n*=10 wells).

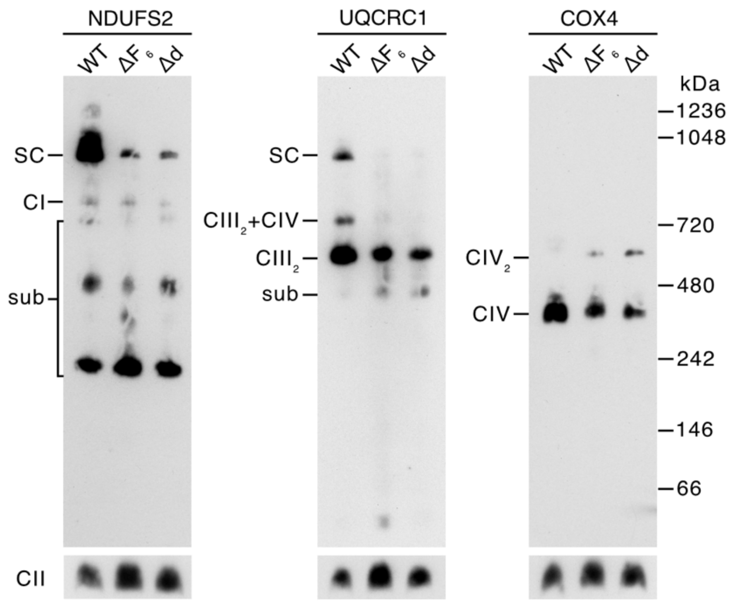

Fig. S6. Effect of deletion of subunits F_6 and d on the assembly of human electron transfer complexes. Mitochondrial membranes from HAP1-WT, -ΔF_6 and -Δd cells were extracted with digitonin (9 g/g protein), and the extracts were fractionated by BN-PAGE. Complexes were detected by western blotting with antibodies against complexes I (NDUFS2), III (UQCRC1) and IV (COX4). Complex II (CII-SDHA) provided a loading control. CI, complex I; $CIII_2$, complex III dimer; CIV, complex IV; CIV_2, complex IV dimer; $CIII_2$+CIV, complex III dimer plus complex IV; SC, supercomplex; sub, subcomplexes. The migration positions of standard proteins are shown on the right.

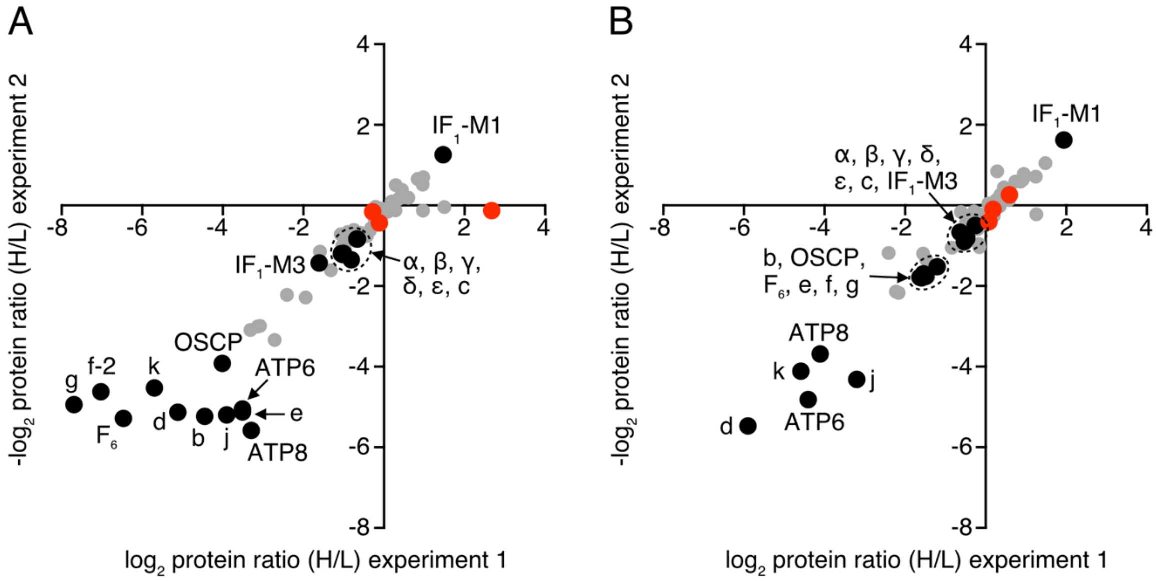

Fig. S7. Relative protein abundances in ATP synthase complexes purified from HAP1-ΔF_6 and HAP1-Δd cells. (*A*) and (*B*), respectively, immunocaptured ATP synthase from HAP1-ΔF_6 and HAP1-Δd cells. Samples were prepared from a 1:1 mixture of HAP1-WT cells with either HAP1-ΔF_6 cells or HAP1-Δd cells, that were differentially SILAC-labelled. The experiments were performed twice with reciprocal SILAC labelling orientations. The protein ratio is derived from a minimum of two peptide ratios from each experiment, except for ATP6 from HAP1-Δd in experiment 2, where the value is from a single peptide ratio. The ratios for proteins obtained in both experiments are plotted as a single point on a scatter plot as the log base 2 value. ●, ATP synthase subunits and forms of IF_1; ●, assembly factors ATPAF2, FMC1 and TMEM70; ●, all other proteins. In (*B*), the data point for Rab11 family-interacting protein 3, RAB11FIP3 (-5.40, 4.59) is outside the axes, in the upper left 'contaminant' quadrant. Protein ratios are given in *SI Appendix* Datasets S1-S4.

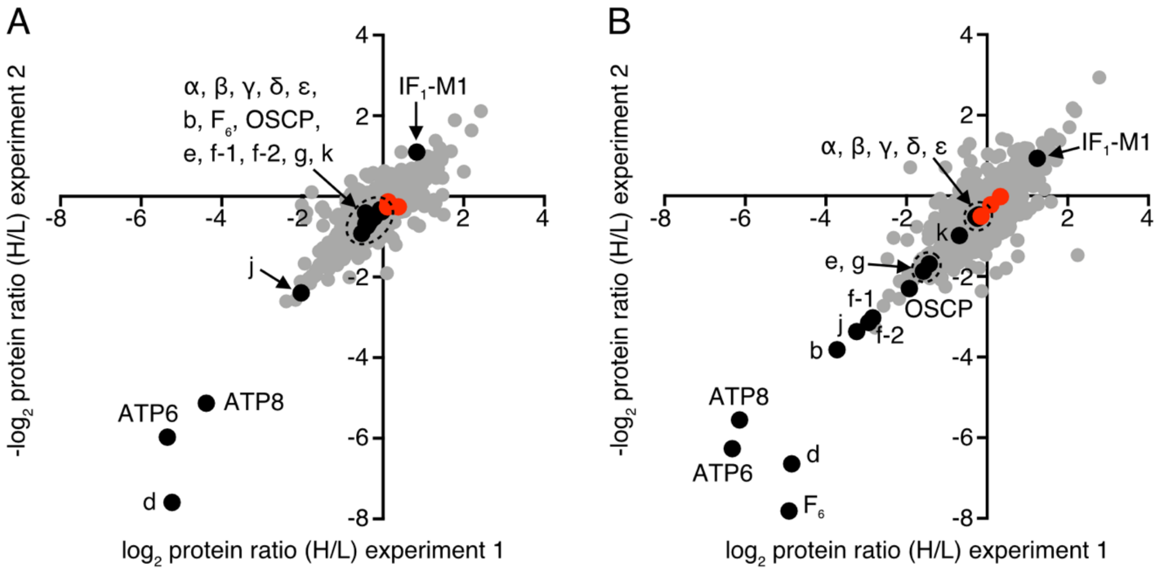

Fig. S8. Effects on protein relative abundance in mitoplast samples of HAP1 cells of the individual deletion of the d-subunit or F_6-subunit of human ATP synthase. Samples were prepared from a 1:1 mixture of HAP1-Δd or HAP1-ΔF_6 cells with HAP1-WT cells that were differentially SILAC-labelled. The experiments were performed twice, using reciprocal SILAC labelling orientations. (*A* and *B*), the ratios for all proteins determined in both of the experimental labelling orientations are plotted as a single point on a scatter plot, as the log base 2 value, for mitoplast samples from HAP1-Δd and HAP1-ΔF_6 cells, respectively. The protein ratio is the median value derived from a minimum of two peptide ratios from each experiment, except for ATP synthase subunit ATP6 in the HAP1-Δd experiment 2, subunit f-2 in both HAP1-Δd experiments, and IF_1-M1 in the HAP1-Δd experiment 1, which only gave one peptide ratio. ●, ATP synthase subunits and the M1 mature forms of IF_1; ●, assembly factors ATPAF1, ATPAF2, and TMEM70; ●, all other identified proteins. Protein ratios are listed in Datasets S5-S8.

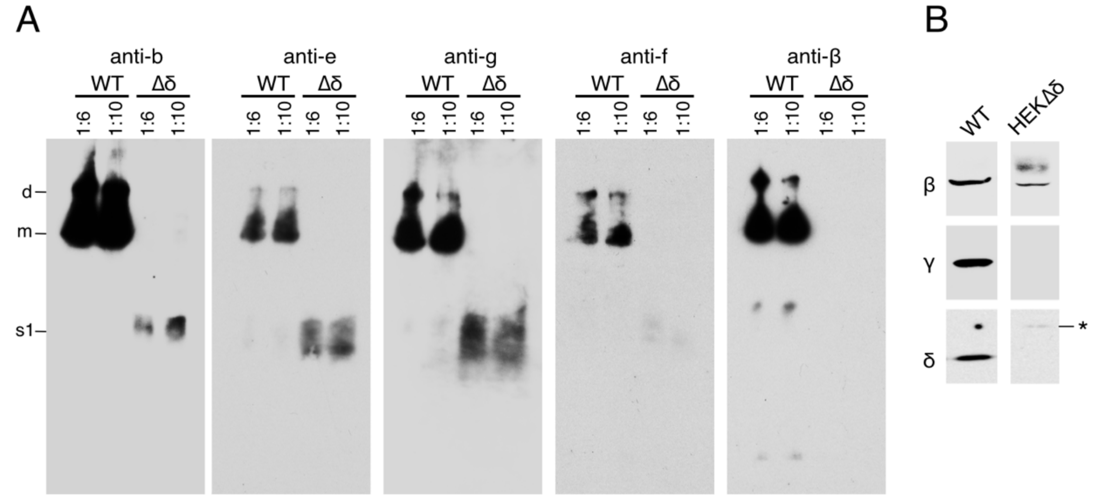

Fig. S9. Vestigial complexes of ATP synthase in HEK293-Δδ cells. (*A*) Fractionation of digitonin extracted complexes from mitoplasts of WT and HEK293-Δδ cells. Samples were extracted with digitonin (6 or 10 g/g protein) and the extracted complexes were fractionated by CN-PAGE, followed by Western blotting and probed with antibodies against subunits b, e, g, f and β (as indicated above the panels). The positions of complexes are shown on the left: d, ATP synthase dimer; m, ATP synthase monomer; s_1, subcomplexes containing subunits b, e, f and g. (*B*) SDS extracted proteins from wild-type (WT) and HEK293-Δδ cells were fractionated by SDS-PAGE and β-, γ- and δ-subunits were detected by western blotting. The HEK293-Δδ cells have a weak band detected with an anti-δ antibody at *ca.* 17-18 kDa, marked with an asterisk.

A

```
                                                    ________________
WT  GGCATCCTGGCGGCCCACGTGCCCACGCTGCAGGTCCTGCGGCCGGGGCTGGTCGTGGTG
Δδ  GGCATCCTGGCGGCCCACGTGCCCACGCTGCAGGTCCTGCGGCCGGGGCTGGTCGTGGTG

    _______^^^
WT  CATGCAGAGGACGGCACCACCTCCAAATACTTTGGTGAGTCCGGTGGAGGGCTGCAGGGC
Δδ  CATG--------------------AAATACTTTGGTGAGTCCGGTGGAGGGCTGCAGGGC


WT  MLPAALLRRPGLGRLVRHARAYAEAAAAPAAASGPNQMSFTFASPTQVFFNGANVRQVDV
Δδ  MLPAALLRRPGLGRLVRHARAYAEAAAAPAAASGPNQMSFTFASPTQVFFNGANVRQVDV

WT  PTLTGAFGILAAHVPTLQVLRPGLVVVHAEDGTTSKYFVSSGSIAVNADSSVQLLAEEAV
Δδ  PTLTGAFGILAAHVPTLQVLRPGLVVVHEILCEQRFHRSERRLFGAVVGRRGRDAGHVGP
                                ********************************
WT  TLDMLDLGAAKANLEKAQAELVGTADEATRAEIQIRIEANEALVKALE
Δδ  GGSQGKLGEGPGGAGGDS------------------------------
    ******************
```

B

```
                                                    ________________
WT  GGCATCCTGGCGGCCCACGTGCCCACGCTGCAGGTCCTGCGGCCGGGGCTGGTCGTGGTG
Δδ  GGCATCCTGGCGGCCCACGTGCCCACGCTGCAGGTCCTGCGGCCGGGGCTGGTCGTGGTG

    _______^^^
WT  CATGCAGAGGACGGCACCACCTCCAAATACTTTGGTGAGTCCGGTGGAGGGCTGCAGGGC
Δδ  CATGCAGAGGACGGCACCACCTCCAAATACTTTGGTGAGTCCGGTGGAGGGCTGCAGGGC
       ▲
    1140 nt

WT  MLPAALLRRPGLGRLVRHARAYAEAAAAPAAASGPNQMSFTFASPTQVFFNGANVRQVDV
Δδ  MLPAALLRRPGLGRLVRHARAYAEAAAAPAAASGPNQMSFTFASPTQVFFNGANVRQVDV

WT  PTLTGAFGILAAHVPTLQVLRPGLVVVHAEDGTTSKYFVSSGSIAVNADSSVQLLAEEAV
Δδ  PTLTGAFGILAAHVPTLQVLRPGLVVVHGILVMRLSKRIFT-------------------
                                *************
WT  TLDMLDLGAAKANLEKAQAELVGTADEATRAEIQIRIEANEALVKALE
Δδ  ---MLDLGAAKANLEKAQAELVGTADEATRAEIQIRIEANEALVKALE
```

Fig. S10. Disruption of the gene for the δ-subunit of human ATP synthase in HEK293 Flp-In^{TM} T-REx^{TM} cells by CRISPR-Cas9. Clonal cells were derived with two differently edited versions of *ATP5F1D*, with in (*A*), a 20 bp deletion, and in (*B*), a 1140 bp insertion (sequence not shown) at the position indicated by the arrowhead. In the upper sections of (*A*) and (*B*), the disrupted DNA sequences are aligned with the wild-type (WT) sequence. The carets above the DNA sequences indicate the PAM (protospacer adjacent motif) sequence for the guide RNA, and solid lines the guide RNA target sequence. In the lower sections, the impact of the deletions on the protein sequence of the δ-subunit are shown. Asterisks indicate changes in the protein sequence arising from the deletions, and the dashes indicate that the protein sequence has been terminated by the introduction of a stop codon. In (*A*) and (*B*), respectively, truncated versions of the δ-subunit were produced consisting of residues 1-88 followed by 50 and 13 unmatched residues. By internal initiation of the translation from four cryptic AUG codons, it is possible that the insertion might lead also to the production of residues 124-168 of the δ-subunit and three unrelated polypeptides (90, 62 and 117 amino acids long, not shown).

Table S1. Target sites for gRNAs employed in the disruption of human genes *ATP5PD* and *ATP5PF*, encoding subunits d and F_6 of human ATP synthase

gRNA	Sequence
ATP5PD-1	CTCTCAGAAACATACTGACC*
ATP5PD-2	TTTCAGGATCCCAAAATGGC
ATP5PF-1	GACGGCTGACCGAATGACAG*
ATP5PF-2	TCCTATACAGAAACTCTTTG

*Antisense sequence used for gRNA

Table S2. Primers employed in the amplification by PCR of regions targeted by gRNAs in *ATP5PD* and *ATP5PF*.

Primer	Sequence
ATP5PD-Forward	TCCCAACTGAAAATTCACCTCAT
ATP5PD-Reverse	AAGATCATCATGAAAATGCAAGGAT
ATP5PF-Forward	TTCCTGTTAGGAGCGGACAG
ATP5PF-Reverse	GAGATGAGCTCAGTGAAGGTCTAAT

Table S3. Sources of Antibodies

Protein	Source	Antibody
ATP synthase δ	Proteintech	14893-1-AP
	ABclonal	A9929
ATP synthase b	Abcam	ab217062
ATP synthase c	Abcam	ab180149, ab181243
ATP synthase F_6	Abcam	ab224139
ATP synthase OSCP	Proteintech	66696-1-Ig
ATP synthase d	Proteintech	17589-1-AP
	In-house	Rabbit polyclonal against recombinant bovine protein
ATP synthase ATP8	Proteintech	26723-1-AP
PHB2	Proteintech	12295-1-AP
SDHB	Sigma	HPA002868
Strep II	Abcam	ab184224
TIM23	Abcam	ab230253

Dataset legends

Dataset S1 (separate file). Proteins identified in SILAC experiments comparing immunopurified ATP synthase from wild-type and HAP1-ΔF_6 cells. This information is the output from Perseus after processing of MaxQuant SILAC peptide pair data. ATP synthase was immunopurified from digitonin solubilized mitoplast material prepared from a 1:1 mixture of wild-type and HAP1-ΔF_6 cells, and digested in-solution with trypsin or chymotrypsin. Experiment 1 refers to heavy isotope labelled HAP1-ΔF_6 cells mixed with light labelled wild-type cells, and experiment 2 vice versa. Perseus processing removed proteins identified in both a decoy database (created in MaxQuant by reversing protein entries) and a contaminant database, experiment 2 ratios were inverted, and the protein ratios rendered base two logarithmic. Only protein groups (listed under 'Protein names') with ratios determined in both experiments are included. ATP5IF1 ratios were manually calculated using data obtained for unique peptides from the N-termini of two mature forms (IF1-M1 and -M3) of the protein (see Dataset S2).

Dataset S2 (separate file). Peptide data for the ATPase inhibitor protein obtained in SILAC experiments comparing ATP synthase from wild-type and HAP1-ΔF_6 cells. This information is obtained from the MaxQuant evidence file after the processing of SILAC peptide pair data. ATP synthase was immunopurified from digitonin solubilized mitoplast material prepared from a 1:1 mixture of HAP1 wild-type and HAP1-ΔF_6 cells. Experiment 1 refers to heavy isotope labelled HAP1-ΔF_6 cells mixed with light labelled wild-type cells, and experiment 2 vice versa. Only peptide data that provided ratio information is included. When fewer than three MULTI-MSMS ratios were obtained ISO-MSMS ratios were also included to determine the protein ratio, represented by the median peptide ratio.

Dataset S3 (separate file). Proteins identified in SILAC experiments comparing immunopurified ATP synthase from wild-type and HAP1-Δd cells. This information is the output from Perseus after processing of MaxQuant SILAC peptide pair data. ATP synthase was immunopurified from digitonin solubilized mitoplast material prepared from a 1:1 mixture of wild-type and HAP1-Δd cells, and digested in-solution with trypsin or chymotrypsin. Experiment 1 refers to heavy isotope labelled HAP1-Δd cells mixed with light labelled wild-type cells, and experiment 2 vice versa. Perseus processing removed proteins identified in both a decoy database (created in MaxQuant by reversing protein entries) and a contaminant database, experiment 2 ratios were inverted, and the protein ratios rendered base two logarithmic. Only protein groups (listed under 'Protein names') with ratios determined in both experiments are included. The protein ratio is derived from a minimum of two peptide ratios from each experiment, except for the MT-ATP6 protein ratio for experiment 2 which is from a single peptide value. ATP5IF1 ratios were manually calculated using data obtained for unique peptides from the N-termini of two mature forms (IF1-M1 and -M3) of the protein (see Dataset S4).

Dataset S4 (separate file). Peptide data for the ATPase inhibitor protein obtained in SILAC experiments comparing ATP synthase from wild-type and HAP1-Δd cells. This information is obtained from the MaxQuant evidence file after the processing of SILAC peptide pair data. ATP synthase was immunopurified from digitonin solubilized mitoplast material prepared from a 1:1 mixture of HAP1 wild-type and HAP1-Δd cells. Experiment 1 refers to heavy isotope labelled HAP1-Δd cells mixed with light labelled wild-type cells, and experiment 2 vice versa. Only peptide data that provided ratio information is included. When fewer than three MULTI-MSMS ratios were obtained ISO-MSMS ratios were also included to determine the protein ratio, represented by the median peptide ratio.

Dataset S5 (separate file). Proteins identified in SILAC experiments comparing mitoplasts from wild-type and HAP1-Δd cells. This information is the output from Perseus after processing of MaxQuant SILAC peptide pair data. Digitonin solubilized mitoplast samples were prepared from a 1:1 mixture of wild-type and HAP1-Δd cells, analyzed by SDS-PAGE and gel sections digested with trypsin. Experiment 1 refers to heavy isotope labelled HAP1-Δd cells mixed with light labelled wild-type cells, and experiment 2 vice versa. Perseus processing removed proteins identified in

both a decoy database (created in MaxQuant by reversing protein entries) and a contaminant database, experiment 2 ratios were inverted, and the protein ratios rendered base two logarithmic. Only protein groups (listed under 'Protein names') with ratios determined in both experiments are included. The protein ratio is derived from a minimum of two peptide ratios from each experiment, except for ATP5MF isoform 2 protein ratio which is from a single peptide value for both experiments, and MT-ATP6 protein ratio for experiment 2 which is from a single peptide value. ATP5IF1 ratios were manually calculated using data obtained for unique peptides from the N-termini of one mature form (IF1-M1) of the protein (see Dataset S6). The IF1-M1 protein ratio for experiment 1 is from a single peptide value.

Dataset S6 (separate file). Peptide data for the ATPase inhibitor protein obtained in SILAC experiments comparing mitoplasts from wild-type and HAP1-Δd cells. This information is obtained from the MaxQuant evidence file after the processing of SILAC peptide pair data. The peptide data is from digitonin protein extracts of mitoplast samples prepared with a 1:1 mixture of HAP1wild-type and HAP1-Δd cells. Experiment 1 refers to heavy isotope labelled HAP1-Δd cells mixed with light labelled wild-type cells, and experiment 2 vice versa. Only peptide data that provided ratio information is included.

Dataset S7 (separate file). Proteins identified in SILAC experiments comparing mitoplasts from wild-type and HAP1-ΔF_6 cells. This information is the output from Perseus after processing of MaxQuant SILAC peptide pair data. Digitonin solubilized mitoplast samples were prepared from a 1:1 mixture of wild-type and HAP1-ΔF_6 cells, analyzed by SDS-PAGE and gel sections digested with trypsin. Experiment 1 refers to heavy isotope labelled HAP1-ΔF6 cells mixed with light labelled wild-type cells, and experiment 2 vice versa. Perseus processing removed proteins identified in both a decoy database (created in MaxQuant by reversing protein entries) and a contaminant database, experiment 2 ratios were inverted, and the protein ratios rendered base two logarithmic. Only protein groups (listed under 'Protein names') with ratios determined in both experiments are included. ATP5IF1 ratios were manually calculated using data obtained for unique peptides from the N-termini of one mature form (IF1-M1) of the protein (see Dataset S8).

Dataset S8 (separate file). Peptide data for the ATPase inhibitor protein obtained in SILAC experiments comparing mitoplasts from wild-type and HAP1-ΔF6 cells. This information is obtained from the MaxQuant evidence file after the processing of SILAC peptide pair data. The peptide data is from digitonin protein extracts of mitoplast samples prepared with a 1:1 mixture of HAP1wild-type and HAP1-ΔF6 cells. Experiment 1 refers to heavy isotope labelled HAP1-ΔF6 cells mixed with light labelled wild-type cells, and experiment 2 vice versa. Only peptide data that provided ratio information is included. When fewer than three MULTI-MSMS ratios were obtained ISO-MSMS ratios were also included to determine the protein ratio, represented by the median peptide ratio.

Dataset S9 (separate file). Proteins identified in SILAC experiments comparing overexpressed tagged-subunit b and overexpressed tagged-subunit j. This information is the output from Perseus after processing of MaxQuant SILAC peptide pair data. Samples were affinity purified from mitoplasts prepared from a 1:1 mixture of Flp-In™ T-REx™ HEK293T-Δδ cells overexpressing either tagged-subunit b or tagged-subunit j, analyzed by SDS-PAGE and gel sections digested with trypsin. Experiment 1 refers to heavy isotope labelled tagged-subunit j cells mixed with light labelled tagged subunit-b cells, and experiment 2 vice versa. Perseus processing removed proteins identified in both a decoy database (created in MaxQuant by reversing protein entries) and a contaminant database, experiment 2 ratios were inverted, and the protein ratios rendered base two logarithmic. Only protein groups (listed under 'Protein names') with ratios determined in both experiments are included. Tagged and endogenous subunit-b or subunit j ratios were manually calculated using data obtained from different migration positions on the gel (see Datasets S10 and S11).

Dataset S10 (separate file). Peptide data for the ATP synthase subunit b protein obtained in SILAC experiments comparing mitoplasts from Flp-In™ T-REx™ HEK293T-Δδ cells

overexpressing either tagged-subunt b or tagged-subunit j. This information is obtained from the MaxQuant evidence file after the processing of SILAC peptide pair data. Samples were affinity purified from digitonin solubilized mitoplast material prepared from a 1:1 mixture of Flp-In™ T-REx™ HEK293T-Δδ cells overexpressing either tagged-subunit b or tagged-subunit j, analyzed by SDS-PAGE and gel sections digested with trypsin. Experiment 1 refers to heavy isotope labelled subunit-j cells mixed with light labelled subunit-b cells, and experiment 2 vice versa. Only peptide data that provided ratio information is included. Gel section 7 corresponds to the tagged-b peptides and gel section 8 those from the endogenous subunit b. When fewer than three MULTI-MSMS ratios were obtained ISO-MSMS ratios were also included to determine the protein ratio, represented by the median peptide ratio.

Dataset S11 (separate file). Peptide data for the ATP synthase subunit-j protein obtained in SILAC experiments comparing mitoplasts from Flp-In™ T-REx™ HEK293T-Δδ cells overexpressing either tagged-subunt b or tagged-subunit j. This information is obtained from the MaxQuant evidence file after the processing of SILAC peptide pair data. Samples were affinity purified from digitonin solubilized mitoplast material prepared from a 1:1 mixture of Flp-In™ T-REx™ HEK293T-Δδ cells overexpressing either tagged-subunit b or tagged-subunit j, analyzed by SDS-PAGE and gel sections digested with trypsin. Experiment 1 refers to heavy isotope labelled subunit-j cells mixed with light labelled subunit-b cells, and experiment 2 vice versa. Only peptide data that provided ratio information is included. Gel section 10 corresponds to the tagged-subunit j peptides and gel section 11 those from the endogenous subunit-j. When fewer than three MULTI-MSMS ratios were obtained ISO-MSMS ratios were also included to determine the protein ratio, represented by the median peptide ratio.

SI References

1. F. A. Ran*, et al.*, Genome engineering using the CRISPR-Cas9 system. *Nat. Protoc.* **8**, 2281-2308 (2013).
2. J. E. Carette*, et al.*, Ebola virus entry requires the cholesterol transporter Niemann-Pick C1. *Nature* **477**, 340-343 (2011).
3. P. Essletzbichler*, et al.*, Megabase-scale deletion using CRISPR/Cas9 to generate a fully haploid human cell line. *Genome Res.* **24**, 2059-2065 (2014).
4. J. Carroll, J. He, S. Ding, I. M. Fearnley, J. E. Walker, Persistence of the permeability transition pore in human mitochondria devoid of an assembled ATP synthase. *Proc. Natl. Acad. Sci. U.S.A.* **116**, 12816-12821 (2019).
5. J. He, J. Carroll, S. Ding, I. M. Fearnley, J. E. Walker, Permeability transition in human mitochondria persists in the absence of peripheral stalk subunits of ATP synthase. *Proc. Natl. Acad. Sci. U.S.A.* **114**, 9086-9091 (2017).
6. J. He*, et al.*, Assembly of the membrane domain of ATP synthase in human mitochondria. *Proc. Natl. Acad. Sci. U.S.A.* **115**, 2988-2993 (2018).
7. J. He*, et al.*, Persistence of the mitochondrial permeability transition in the absence of subunit c of human ATP synthase. *Proc. Natl. Acad. Sci. U.S.A.* **114**, 3409-3414 (2017).
8. P. Skehan*, et al.*, New colorimetric cytotoxicity assay for anticancer-drug screening. *J. Natl. Cancer Inst.* **82**, 1107-1112 (1990).
9. S. E. Ong*, et al.*, Stable isotope labeling by amino acids in cell culture, SILAC, as a simple and accurate approach to expression proteomics. *Mol. Cell. Proteomics* **1**, 376-386 (2002).
10. J. He*, et al.*, Human C4orf14 interacts with the mitochondrial nucleoid and is involved in the biogenesis of the small mitochondrial ribosomal subunit. *Nucleic Acids Res.* **40**, 6097-6108 (2012).
11. V. F. Rhein*, et al.*, Human METTL20 methylates lysine residues adjacent to the recognition loop of the electron transfer flavoprotein in mitochondria. *J. Biol. Chem.* **289**, 24640-24651 (2014).
12. P. Klement, L. G. Nijtmans, C. Van den Bogert, J. Houstěk, Analysis of oxidative phosphorylation complexes in cultured human fibroblasts and amniocytes by blue-native-

electrophoresis using mitoplasts isolated with the help of digitonin. *Anal. Biochem.* **231**, 218-224 (1995).
13. H. Schägger, G. von Jagow, Blue native electrophoresis for isolation of membrane protein complexes in enzymatically active form. *Anal. Biochem.* **199**, 223-231 (1991).
14. I. Wittig, M. Karas, H. Schägger, High resolution clear native electrophoresis for in-gel functional assays and fluorescence studies of membrane protein complexes. *Mol. Cell. Proteomics* **6**, 1215-1225 (2007).
15. V. F. Rhein, J. Carroll, S. Ding, I. M. Fearnley, J. E. Walker, NDUFAF7 methylates arginine 85 in the NDUFS2 subunit of human complex I. *J. Biol. Chem.* **288**, 33016-33026 (2013).
16. M. Wilm*, et al.*, Femtomole sequencing of proteins from polyacrylamide gels by nano-electrospray mass spectrometry. *Nature* **379**, 466-469 (1996).
17. J. Cox, M. Mann, MaxQuant enables high peptide identification rates, individualized p.p.b.-range mass accuracies and proteome-wide protein quantification. *Nat. Biotechnol.* **26**, 1367-1372 (2008).
18. J. Cox*, et al.*, Andromeda: a peptide search engine integrated into the MaxQuant environment. *J. Proteome Res.* **10**, 1794-1805 (2011).
19. G. Xu, S. B. Shin, S. R. Jaffrey, Global profiling of protease cleavage sites by chemoselective labeling of protein N-termini. *Proc. Natl. Acad. Sci. U.S.A.* **106**, 19310-19315 (2009).
20. A. S. Vaca Jacome*, et al.*, N-terminome analysis of the human mitochondrial proteome. *Proteomics* **15**, 2519-2524 (2015).
21. M. R. Dyer, J. E. Walker, Sequences of members of the human gene family for the c subunit of mitochondrial ATP synthase. *Biochem. J.* **293**, 51-64 (1993).
22. W. L. Yan, T. J. Lerner, J. L. Haines, J. F. Gusella, Sequence analysis and mapping of a novel human mitochondrial ATP synthase subunit 9 cDNA (ATP5G3). *Genomics* **24**, 375-377 (1994).
23. T. B. Walpole*, et al.*, Conservation of complete trimethylation of lysine-43 in the rotor ring of c-subunits of metazoan adenosine triphosphate (ATP) synthases. *Mol. Cell. Proteomics* **14**, 828-840 (2015).
24. S. Tyanova*, et al.*, The Perseus computational platform for comprehensive analysis of (prote)omics data. *Nat. Methods* **13**, 731-740 (2016).
25. J. Cox, M. Mann, Quantitative, high-resolution proteomics for data-driven systems biology. *Annu. Rev. Biochem.* **80**, 273-299 (2011).
26. T. E. Spikes, M. G. Montgomery, J. E. Walker, Structure of the dimeric ATP synthase from bovine mitochondria. *Proc. Natl. Acad. Sci. U.S.A.* **117**, 23519-23526 (2020).
